# Enhancement of the structure, solar cells and vibrational studies of undoped CuCr2O4 and La-doped CuCr2O4 semiconductor compounds

**DOI:** 10.1016/j.heliyon.2022.e09233

**Published:** 2022-04-01

**Authors:** G. Rajeswari, N. Prabavathi, P. Tamizhdurai, A. Prakasam, G. Kumar

**Affiliations:** a Department of Physics, Sri Sarada College for Women (Autonomous), Salem, 636016, India; b Department of Chemistry, Dwaraka Doss Goverdhan Doss Vaishnav College (Autonomous), E.V.R. Periyar Road, Arumbakkam, Chennai, Tamil Nadu, 600 106, India; c Department of Physics, Thiruvalluvar Government Arts College, Rasipuram, Tamil Nadu, 637401, India; d Department of Chemistry, Presidency College (autonomous), Chennai, Tamil Nadu, 600 005, India

**Keywords:** Chemical synthesis, Nanostructured materials, CuCr_2_O_4_, Lanthanum, Inorganic materials

## Abstract

In the present paper, we report the successful synthesis of spinel-type of CuCr_2_O_4_ and La doped CuCr_2_O_4_ semiconductor nanoparticles by a microwave method. Starting with the precursor complex, this technique includes the creation of homogenous solid intermediates, which reduces atomic diffusion pathways during the microwave process. CuCr_2_O_4_ and La doped CuCr_2_O_4_ were characterized by the following analytical methods for instance X-ray diffraction (XRD), transmission electron microscopy (TEM), Ultraviolet–visible (UV–Vis) spectroscopy and Fourier transform infrared spectroscopy (FTIR). The results demonstrated that modifying the precursor had a significant impact on the size, solar cell size, as well as reaction period of synthesizing CuCr_2_O_4_ and La doped CuCr_2_O_4_. The impacts of precursors on the morphological and structural characteristics of CuCr_2_O_4_ and La doped CuCr_2_O_4_ were examined for the first time in this publication.

## 1 Introduction

In the past few years, Rare-earth oxides have been employed as extensively catalysts, magnets, superior luminescent devices, and other applications in accordance with the chemical, optical, and electronic properties resulting from their 4f electrons [1]. Because nanocrystalline materials have been shown to possess outstanding chemical as well as physical characteristics, for instance improved phase uniformity and improved sinterability at low temperatures, much work has been put into synthesizing and characterizing of metal oxide nano-ceramics. Spinel-type mixed oxides (AB_2_O_4_) are widely recognized among the many inorganic solids for their outstanding characteristics [2, 3].

Generally, the combustion-based technique is a simple and comfortable way for producing a wide range of nanomaterials, catalysts, and innovative ceramics. Furthermore, solution-based techniques for the production of copper aluminate nanoparticles have been described, including the sol-gel [4], polymeric precursor method [5], and combustion method [6].

Though, materials synthesis through microwave approach has obtained significant compared to conventional approach recently, because of the fact that microwave interact at the molecular stage with the reactants, as a result the electromagnetic energy transmitted and changed into heat by fast kinetics by molecules movement. As a result, the nanoparticles formed in a couple of minutes with various morphologies and the phase formed early. Moreover, microwave sintered via conventional ways can produce smaller particle sizes as well as better homogeneous microstructures since the heat is produced inside within the material by interaction of microwave–material rather than heat coming from outside sources [7, 8, 9]. In comparison to the other ways, the microwave method is a practical and appealing method for preparing CuCr_2_O_4_ and La doped CuCr_2_O_4_ due to ultrafine with highest purity powders can be generated at quite low temperatures.

In recent times, thus, the selection of properties to control the semiconductors' shapes and sizes has become one of the most difficult issues currently being challenged by material researchers. In particular, significant works have already been made in recent years to improve the synthesis methods for the preparation of CuCr_2_O_4,_ and La doped CuCr_2_O_4_ semiconductor nanostructures. Nonetheless, even now there is a shortage of study on optical characteristics, which have a significant impact on its contributions in microelectronic areas, as stated in the research article [10, 11, 12, 13]. According to that, we have synthesized pristine CuCr_2_O_4,_ and different concentrations of La doped CuCr_2_O_4_ nanostructures. The primary objective of the present research is to examine and authenticate the influence of La concentration on CuCr_2_O_4,_ and La doped CuCr_2_O_4_ semiconductors’ structural, optical, and solar cells properties.

## 2 Experimental method

### 2.1 Synthesis of CuCr_2_O_4_ and La doped CuCr_2_O_4_

The following steps is an example of a common synthesis procedure for CuCr_2_O_4_ and La doped CuCr_2_O_4_ nanoparticles. In the first step, solutions of Cr(NO_3_)_3_.9H_2_O and Cu(NO_3_)_2_·6H_2_O were prepared in a separate beaker and followed by the two salt solution combined into a single mixture. Afterwards, 0.5 M concentration of La(NO_3_)_3_.6H_2_O (6 ml) was introduced into the aforementioned mixture (for La doped CuCr_2_O_4_ nanoparticles synthesis). In addition, 0.6 M concentration of carbamide solution (6 ml) was added to the aforementioned solution drop by drop. The final mixture was then placed for 20 min in a household microwave oven (Model number and manufacturer: 20SC2 and IFB) to be heated by the microwave. The microwave oven has a capacity of 1200 Watts microwave energy with a microwave frequency of 2450 MHz. During microwave heating, the final uniform mixture began to boil, followed by gases released during evaporation. When a chemical combination reaches the stage of automatic combustion, it starts vaporizing followed by the solid is formed at that moment. The resultant substance was thoroughly cleaned by ethyl alcohol before being dehydrated at about 180 °C for 1 h. The pristine CuCr_2_O_4_ (0.00) and La-doped CuCr_2_O_4_ (0.01–0.03) samples were designated as (a) and (b) to (d).

### 2.2 Characterization

An X-ray diffractometer (Brand: Rigaku) with a CuK light source was used to investigate CuCr_2_O_4_ and La-doped CuCr_2_O_4_ samples for obtaining the X-ray diffraction patterns. Images of high-resolution scanning electron microscopy (HR-SEM) acquired using a Philips XL30 ESEM instrument. HR-SEM is linked to energy-dispersive X-ray spectroscopy. Pictures of transmission electron microscopy with higher resolution (HR-TEM) were obtained using a transmission electron microscope with a 200 kV accelerating voltage (Manufacturer and Design: Philips EM 208). The electrocatalytic activity of assynthesized CuCr_2_O_4_ samples was investigated using the exchange current density. At room temperature, the *J–V* (current density-voltage) of dye-sensitized solar cells (DSSCs) was recorded by PEC-L01 Portable Solar Simulator with 100 mWcm^−2^ (Maked by and Model: Peccell, Japan and PEC-L01) and 1.5 AM illumination.

## 3 Results and discussion

### 3.1 Crystal structure analysis

**Figure 1:**
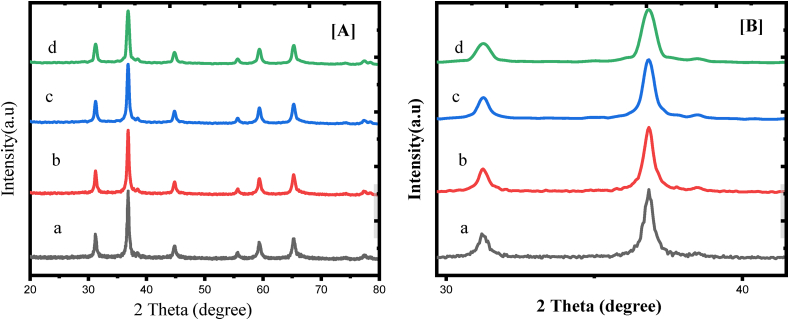
A) X-ray diffraction pattern and B) Theta value (34–43) of a) undoped CuCr_2_O_4_ b) 0.01, c) 0.02, and d) 0.03 La doped CuCr_2_O_4_.

The crystal structure of the studied samples undoped CuCr_2_O_4_ and La doped CuCr_2_O_4_ (0.01, 0.02 and 0.03%) doped CuCr_2_O_4_ are given in Figure 1A. Crystal structure of each individual specimen identified by X-ray difference analysis. The revealed diffraction peaks match with the undoped CuCr_2_O_4_ and La doped CuCr_2_O_4_ standard patterns, which can be in agree well with the relevant JCPDS (Joint Committee on Powder Diffraction Standards) card: 89–6615. In the beginning, the tetrahedral sites occupied by La ions (up to x = 0.01). But, the La ions became dispersed in the octahedral and tetrahedral sites (Figure 1B). Although, there is a shift in the direction of a low angle observed in every intense peak by doping. This peak shifting clearly shows an effect of lattice expansion because of the successful La ions substitution in the crystal lattice. Peak intensity changes often occur as a result of differences in the content of La, Cu and Cr in spinel type oxides. The indexing of the peaks is done by making use of d-spacing values of the samples. A very high peak alongside *hkl* (311) value Table 1, displays the samples crystal structure. The migration of Cu, Cr and La ions at the octahedral and tetrahedral location in the oxides creates a difference in the intensity of the peaks [14, 15]. In addition to the shift, it was found that the intensity of the peaks was found to decrease and the full-width-half-maximum (FWHM) was increased by the Cu, Cr and La doping, which is given an explanation by the decrease of crystal size by the addition of transition metal atoms [16]. As the inter-atomic distance between the particles decreases, the cell volume and lattice parameter increase with increasing doping concentration. The size of the crystal decreases, which indicates that the crystallization steps is further improving [17, 18, 19].

**Table 1:** Calculated parameters for undoped CuCr_2_O_4_ and La doped CuCr_2_O_4_ from 2Theta 37-36 (***hkl*** plane 311) using XRD analysis.

samples	2Ɵ (degrees)	FWHM,β (degrees)	d-spacing (nm)	lattice constant, a (nm)	Crystallite size, D (nm)
a	37.06	0.080	0.2737	0.5498	30.45
b	36.96	0.085	0.2738	0.5496	30.12
c	36.76	0.088	0.2739	0.5493	30.01
d	36.66	0.093	0.2740	0.549	29.76

In general, by employing the Debye-Scherrer equation and with the X–ray line broadening approach, mean crystallite size (L) of synthesized nanomaterials calculated (Eq. (1)) [20].

(1) $$L=\frac{0.89\lambda }{\beta \;\cos\;\theta }$$

In the Debye-Scherrer formula (Eq. (1)), the L is meant for average crystal size (Å) and the λ denotes the X-ray wavelength source correspondingly. The β depicts the FWHM (full width at half maximum) and the θ (radians) depict observed peaks’ diffraction angle respectively. To determine the instrumental broadening by decoupling these contributions, a diffraction pattern of line expansion is required for a standard material for example silicon. The XRD data such as crystallite size, lattice constant, d-spacing, FWHM, and 2θ (degrees) of undoped CuCr_2_O_4_ and La doped CuCr_2_O_4_ were summarized in Table 1. The undoped CuCr_2_O_4_ and La doped CuCr_2_O_4_ nanoparticles average crystallite sizes are found from 34 to 21.87 nm.

### 3.2 Formation energies

In the lattice, the Schottky defects are generated by component ions transfer into the surface from their bulk positions. At the same time, the Frenkel defects are sets of interstitials and vacancies of the similar type of ion. The octahedral or tetrahedral interstitial sites are considered in a normal spinel lattice. Though, the most persistent amounts of intermediates in these oxides (CuCr_2_O_4_ and La doped CuCr_2_O_4_) are the intermediates between the traditional intermediate sites. The Schottky and Frenkel, formation energies given in Table 2 have been obtained from defect energy calculations of vacancies, and interstitials, in the lattice. In CuCr_2_O_4_, the Schottky defect is pure (*V*_Cu_+ 2*V*_Cr_ + 4*V*_O_); doped (*V*_Cu_–_La_ + 2*V*_Cr_–_La_+4*V*_O_); the Frenkel defect pairs are pure (*V*Cu + Cu*i*), (*V*Cr + Cr*i*), and (*V*O + O*i*); doped (*V*Cu + Cu*i*), and (*V*Cr + Cr*i*), (*V*La + La*i*)and (*V*O + O*i*). As shown in Table 2, the lowest formation energy per defect is for the pair in the cation sublattice, which is followed by the Schottky and Frenkel pairs. In addition, signal of a La-related phase, such as, binary copper chromites, doped phase, was identified, or it may be that the CuCr_2_O_4_ peaks for La-doped nanoparticles is very small and difficult to distinguish. This implies that the La ions replaced Cu sites without significantly changing the crystal structure of copper chromites. This can be accounted for by the fact that the ionic radius of the La^3+^ (1.13 A), was very close to that of Cu^2+^ (0.73 Å) and Cr^3+^ (ionic radius: 0.52 Å). As a result, La may easily enter the copper chromite crystal lattice [21].

**Table 2:** Formation Energies (per defect) in CuCr_2_O_4_ and La doped CuCr_2_O_4_.

Defect	Formation energy (eV)
Frenkel	----
Cu	3.1
Cr	5.2
O	4.9
La	6.1
Schottky	2.8

### 3.3 Functional group analysis

**Figure 2:**
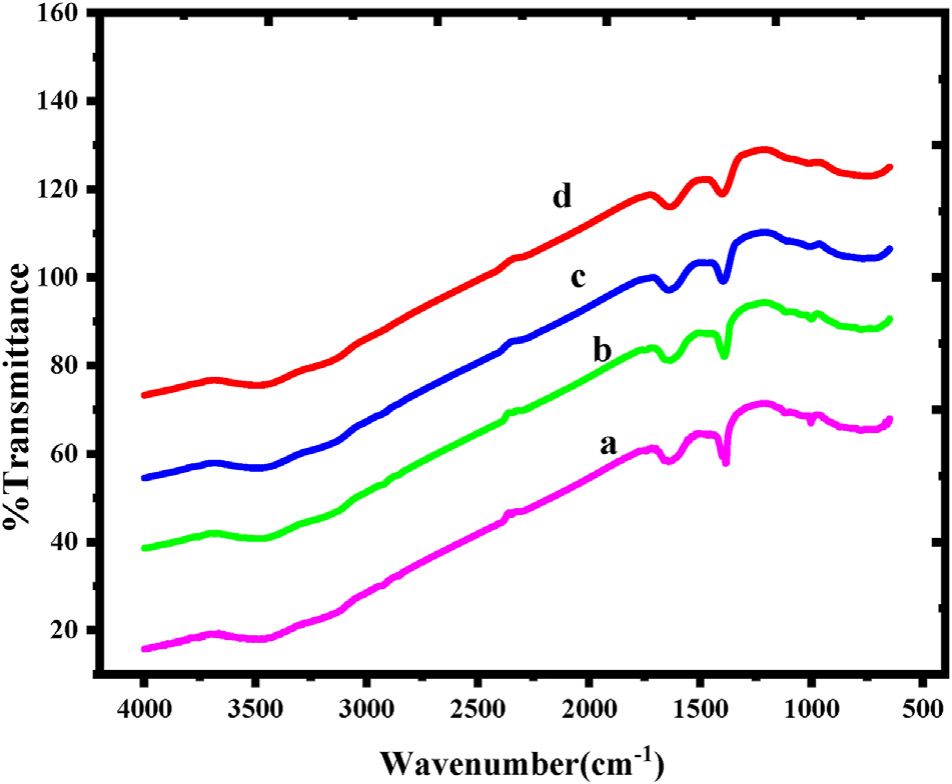
FT-IR spectra of a) undoped CuCr_2_O_4_ b) 0.01, c) 0.02, and d) 0.03 La doped CuCr_2_O_4_.

The analyses of the functional groups are carried out by fourier transform infrared spectra (FT-IR) analysis (Figure 2 (a-d)). The common bandwidth about 3460 and 1620 cm^−1^ can be noticed in this figure for the vibration mode of chemically linked hydroxyl groups and the deformation vibration of water molecules, respectively [22]. In all situations, common bands exist, for examples the wide –OH group centered about at 1570 cm^−1^, 1380 cm^−1^, and 3500 cm^−1^, which are assigned to the stretching vibration of water molecule. The strong absorption band at 2350 cm^−1^ is allotted to CO_2_ molecules’ resulting from stretching vibration mode. The Cu–OH, Cr–OH and La–OH deformation mode vibrations are assigned based on the absorption between 2000 to 900 cm^−1^, which is common in copper chromites. Furthermore, the strong multiple bands in the range 2000-900 cm^−1^ are mostly caused by the deformation modes of hydrogen-bonded –OH groups. The shifting of absorption peaks from higher to lower wave numbers indicates the development of copper chromites. Because of their large surface area, these materials absorb water as well as carbon dioxide from the atmosphere quickly [23]. The existence of octahedral (OH) and tetrahedral (Td) coordinated Metal–Oxygen bonding is ascribed to metal ions dispersed in two distinct habitats in the spinel at 521 and 730 cm^−1^, respectively. and the development of two broad bands may be very clearly attributed to phase formation. The broad absorption bands of CuCr_2_O_4_ and La doped CuCr_2_O_4_ indicated the spinel nature of all compositions. The Metal-Oxygen stretching frequencies between 500-920 cm^−1^ are assigned to the Cr–O, Cu–O–Cr, and Cu–O–Cr–La bonds vibrations for the copper chromites. Copper chromites spinels are distinguished by two wide bands found at 917 and 521 cm^−1^ [24].

### 3.4 High resolution transmission electron microscopy (HR-TEM) studies

**Figure 3:**
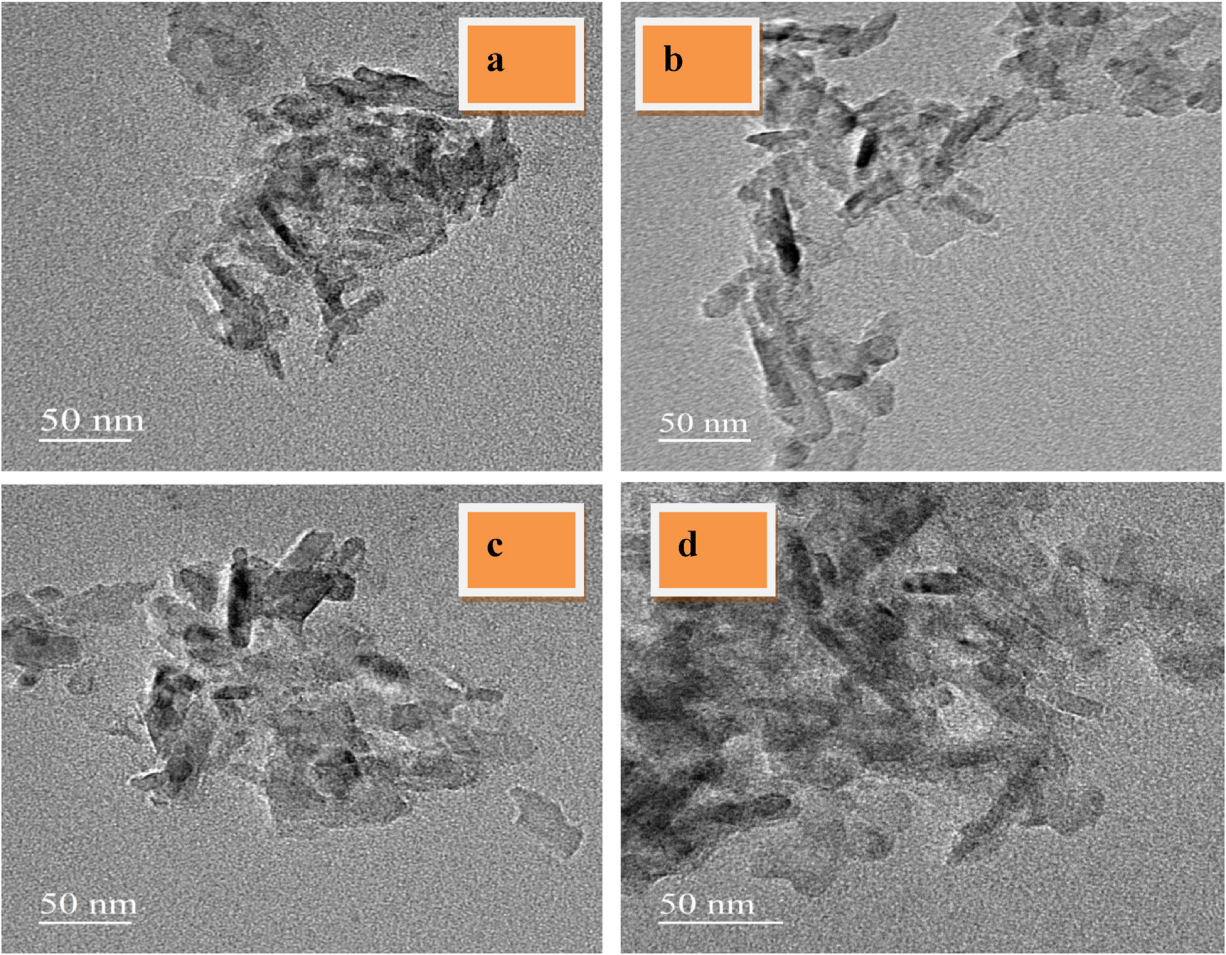
HR-TEM images of a) undoped CuCr_2_O_4_ b) 0.01, c) 0.02, and d) 0.03 La doped CuCr_2_O_4_.

**Figure 4:**
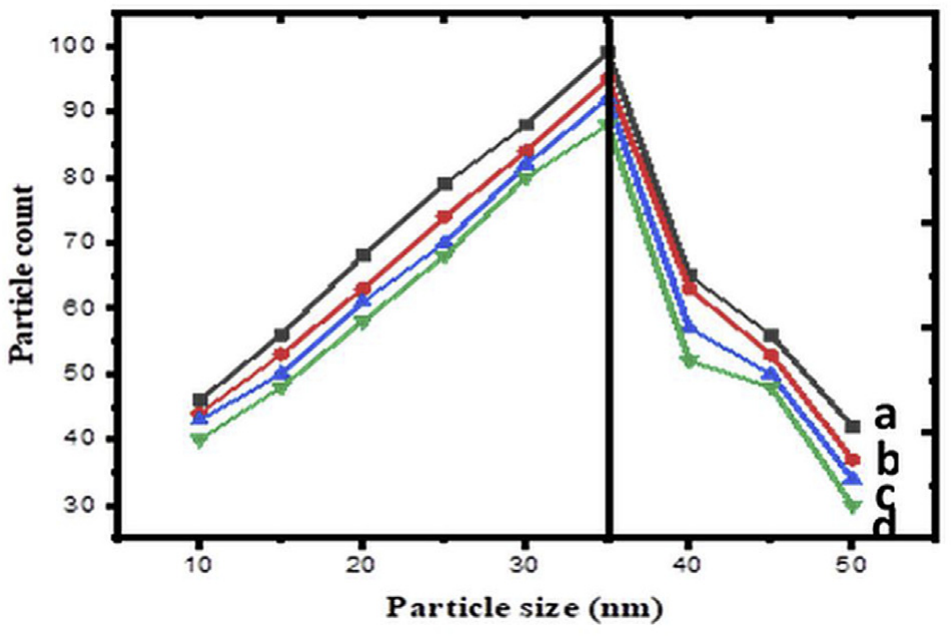
Particle size distribution of a) undoped CuCr_2_O_4_ b) 0.01, c) 0.02, and d) 0.03 La doped CuCr_2_O_4_.

Figure 3(a-d) shows the TEM images of CuCr_2_O_4_ and La doped CuCr_2_O_4_ samples. The assynthesized products are aggregated smaller nanoparticles having a spherical form, as observed from the images. The nanoparticles size determined from the XRD pattern agrees well with TEM investigations, which show a size between 41-25 nm. As a result, we can observe that the big spheres are made up of some tiny nanoparticles with a diameter of around 20 nm. The nucleation clusters are then converted into CuCr_2_O_4_ and La doped CuCr_2_O_4_, which aggregate to create huge spherical aggregated sheets to decrease surface energy. The size of nanoparticles determined from XRD diffraction patterns is consistent with TEM investigations. Of course, the precise method of mechanism has to be explored further. The particle size distribution (histogram) of the nanoparticles was prepared for samples (1–4) (Figure 4). When particle sizes are taken into consideration, the starting and ending points are between 41–25 nm. The centre of this size distribution is 35 nm.

### 3.5 Surface characteristics

We measured the specific surface area of the samples to better understand the surface properties of materials prepared via soft chemical approaches. Surface and pore properties of the CuCr_2_O_4_ nanostructures are presented in Table 3. The combination of a high pore volume and a small pore diameter suggests that the **copper chromites** specific surface (cm^3^/g) area are related with deep pores, while the average pore radius (Å) and total pore volume (cm^3^/g) are made up of relatively shallow pores. The samples have an average pore diameter because of the growth of interstitial pores caused by the mixing of metal oxides to form CuCr_2_O_4_ and La doped CuCr_2_O_4_. Thus, by the microwave method, BET surface area of nano CuCr_2_O_4_ and La doped CuCr_2_O_4_ is improved together with the lowering of the mean pore diameter. This soft chemical technique may be used to create catalysts with large surface areas and narrow crystal size distributions. Thus, it is expected that La doped CuCr_2_O_4_ would exhibit better catalytic activity.

**Table 3:** BET surface area, average pore diameter, pore volume and their crystallite size (nm) of CuCr_2_O_4_ and La doped CuCr_2_O_4_ prepared by microwave method.

Catalyst	A	B	C	D
S_BET_ (m^2^/g)	37.87	41.87	50.14	53.54
R_p_ (Å)	20.20	18.16	19.11	15.89
V_p_ (cm^3^/g)	0.0731	0.0837	0.0956	0.0993
crystallite size (nm)	34.23	30.65	25.34	21.31

### 3.6 Diffuse reflectance spectral studies

**Figure 5:**
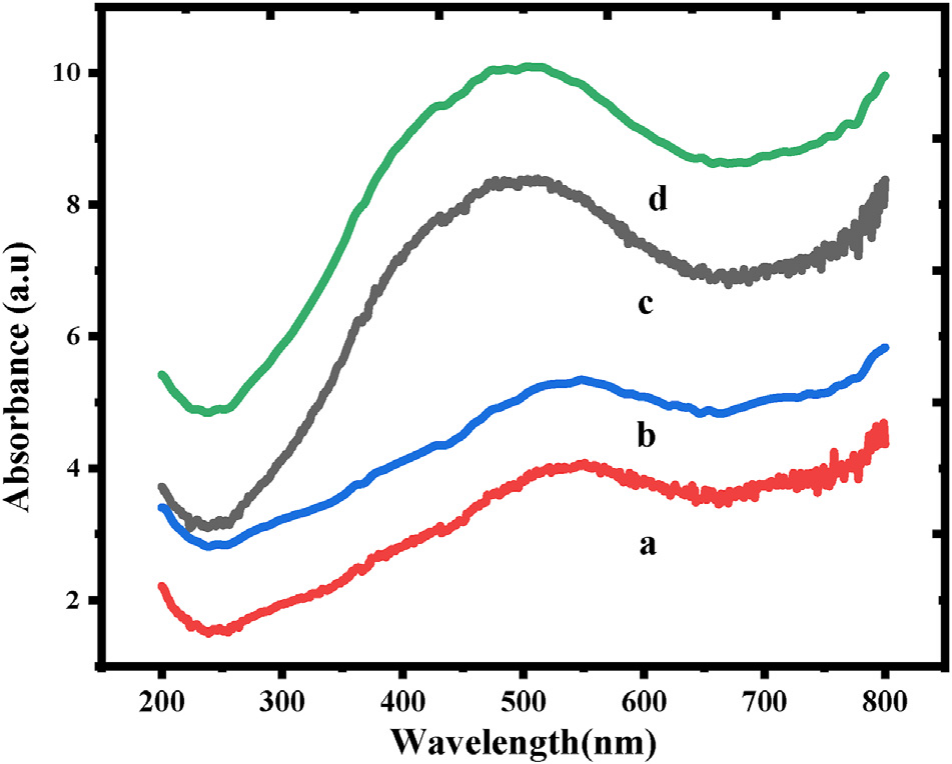
Diffused reflectance UV–Vis spectra of a) undoped CuCr_2_O_4_ b) 0.01, c) 0.02, and d) 0.03 La doped CuCr_2_O_4_.

By analyzing UV–Vis spectra, the optical characteristics of as-prepared CuCr_2_O_4_ and La doped CuCr_2_O_4_ nanocrystals were investigated further quantitatively. The absorption in the visible and UV regions was rather significant, with a maximum wavelength of 200–800 nm (Figure 5). All spectra show a two-structured wide absorption that ranges between 400-600 nm, with the highest spanning from 345 to 550 nm. The **copper chromites** spinel samples display not only the two usual adsorption bands owing to Cu^2+^ ions in tetrahedral and octahedral symmetry, with upper limit situated in the wavelength between 400 to 600 nm, in addition to it a band at 460 nm. In comparison to the tetrahedral band, the octahedral absorption band of pristine spinel is significantly more powerful and symmetric. Similar investigations have linked this behavior to the surface segregation of CuCr_2_O_4_ and La doped CuCr_2_O_4_, with the bands changing to an additional asymmetric signal with a lower intensity. Small particles can only exist in potential wells with a small lateral dimension. The energy variation among the unbound electron and the conduction band allows us to calculate their energy levels. The energy levels approach the more distinct atomic levels as particle size decreases. The reduction of absorbance might have taken place due to agglomeration of nanoparticles [25, 26, 27].

### 3.7 DSSCs analysis

**Figure 6:**
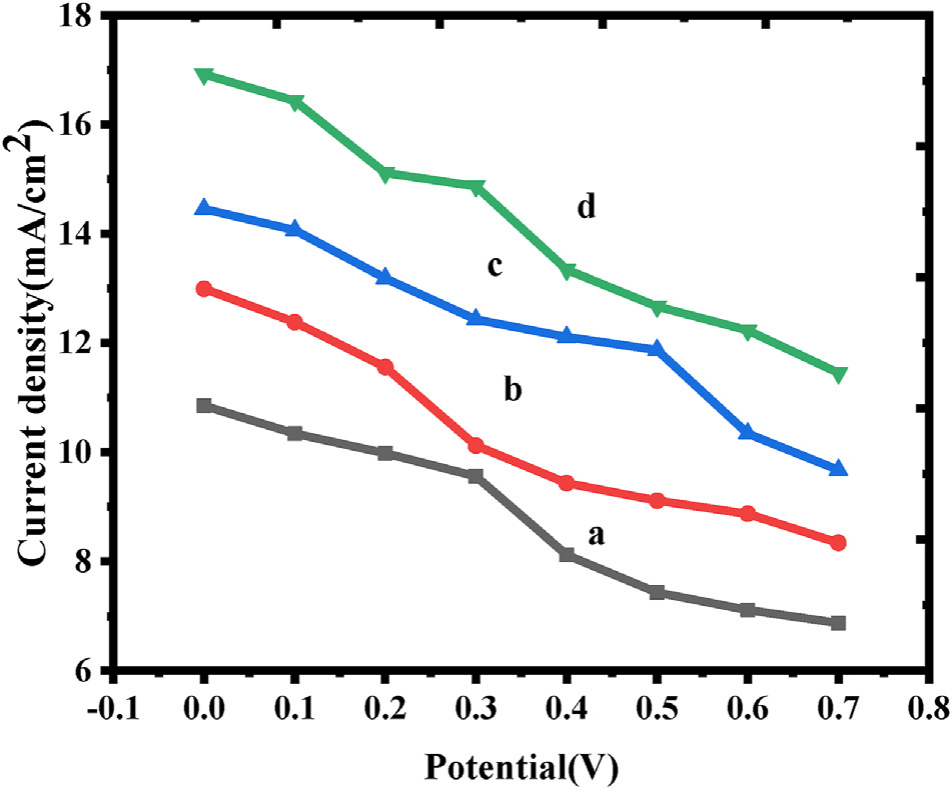
The *J*–*V* curves of DSSCs fabricated with solar cells of a) undoped CuCr_2_O_4_ b) 0.01, c) 0.02, and d) 0.03 La doped CuCr_2_O_4_.

DSSCs have increased in importance because of their low cost of design, simple production procedures, as well as relatively good power conversion efficiency (PCE). Figure 6 depicts the *J–V* curves of the CuCr_2_O_4_ and La doped CuCr_2_O_4_ constructed DSSCs. Table 4 contains detailed data on all photovoltaic characteristics, including the photovoltaic efficiency (PCE), short-circuit photocurrent density (Jsc), open-circuit voltage (Voc), and also the fill factor (FF) [28]. DSSC made from the pristine CuCr_2_O_4_ has the lowest photovoltaic efficiency of 6.05 %, the higher short-circuit photocurrent density of 14.02 mAcm^−2^, and the lowest fill factor of 0.60 respectively. The photovoltaic efficiency was substantially smaller compared to that of DSSCs based on pure oxides (7.10 %). On the other hand, doped oxides displayed a highest short-circuit photocurrent density of 17.63 mAcm^−2^ and the highest fill factor of 0.77 ± 0.06. The increased photoelectric efficiency of the La doped CuCr_2_O_4_ electrode was mostly due to the morphology of La doped CuCr_2_O_4_ and synergistic effect of a significantly dispersive active center [29].

**Table 4:** Photocurrent density (*J*sc), open-circuit voltage (*V*oc), fill factor (*FF*) and PCE are collected CuCr_2_O_4_ and La doped CuCr_2_O_4_ prepared by microwave method.

Samples	*V*oc(V)	*J*sc(mA/cm2)	*FF*	PCE (%)
a	0.702 ± 0.004	10.02 ± 0.21	0.59 ± 0.01	10.05 ± 0.01
b	0.703 ± 0.006	12.16 ± 0.23	0.63 ± 0.02	14.32 ± 0.03
c	0.724 ± 0.007	14.74 ± 0.76	0.62 ± 0.04	18.95 ± 0.05
d	0.748 ± 0.009	16.52 ± 0.34	0.67 ± 0.06	26.65 ± 0.09

## 4 Conclusion

Undoped CuCr_2_O_4_ and La doped CuCr_2_O_4_ hexagonal morphology nanoparticles were synthesized by employing a microwave approach. The impact of La doped CuCr_2_O_4_ with respect to structural, magnetic, and in addition optical properties were investigated. Effect of doping on the crystallinity, particle size, solar cells, and DRS spectra as well as optical properties towards the prepared La doped CuCr_2_O_4_ nanoparticles were noticed. TEM assessment provided in which the forming of hexagonal-like morphological structures within the nanoscale for particle sizes are in matches with the XRD results.

## Declarations

### Author contribution statement

G. Rajeswari: Performed the experiments; Wrote the paper.

N. Prabavathi: Conceived and designed the experiments; Wrote the paper.

P. Tamizhdurai: Analyzed and interpreted the data; Wrote the paper.

A. Prakasam: Conceived and designed the experiments; Analyzed and interpreted the data.

G. Kumar: Performed the experiments; Contributed reagents, materials, analysis tools or data.

### Funding statement

This research did not receive any specific grant from funding agencies in the public, commercial, or not-for-profit sectors.

### Data availability statement

The authors do not have permission to share data.

### Declaration of interests statement

The authors declare no conflict of interest.

### Additional information

No additional information is available for this paper.
